# 

**Published:** 1960-03

**Authors:** 


### MEDICAL JOURNAL OF THE SOUTH-WEST

*(Incorporating the Bristol Medico-Chirurgical Journal)*

Established 1883

VOL. 75 (ii) March 1960 NO. 276

### *Contents*

DOMESTIC HAZARDS WITH NEUROLOGICAL SIGNIFICANCE . . . . . *A. M. G. Campbell* 23

IGNAZ PHILIPP SEMMELWEIS AND HIS TIMES . . . . *Frederick Kohn* 29

"RENAL COMPLICATIONS OF DIABETES MELLITUS" . . *Professor T. F. Hewer* 37

NOTES AND NEWS . . . . . . . 41

EXAMINATION RESULTS . . . . . . 41

APPOINTMENTS . . . . . . . . 41

*PUBLISHED QUARTERLY*

*ANNUAL SUBSCRIPTION* 16/- *POST FREE*

PUBLISHED UNDER THE AUSPICES OF THE
BRISTOL MEDICO-CHIRURGICAL SOCIETY

### *Editorial Committee*

Dr. J. E. Cates, Department of Medicine, Bristol Royal Infirmary. *Editor.*
Dr. N. J. Brown, Southmead Hospital, Bristol. *Assistant Editor.*
Dr. J. Macrae, Ham Green Hospital.
Dr. R. C. Wofinden, Central Clinic, Bristol.
Mr. A. E. S. Roberts, Medical Library, University of Bristol. *Secretary.*

### *Local Correspondents*

*Bath* . . Mr. A. D. Bateman, 3 The Circus, Bath.
*Exeter* . . Dr. L. N. Jackson, Mount Jocelyn, Crediton, Devon.
*Gloucester* . Dr. R. F. Jarrett, 9 College Green, Gloucester.
*Plymouth* . Dr. C. R. Croft, Lexden, Hartley Av., Plymouth.
*Taunton* . Dr. M. McAlley, 1 The Crescent, Taunton.
*Torquay* . Dr. A. Robinson Thomas, 20 Devon Square, Newton Abbot, Devon.
*Truro* . . Dr. F. D. M. Hocking, St. Andrews, Perranporth, Cornwall.
*Yeovil* . . Mr. J. A. Vere Nicoll, Knapp House, Yeovil, Somerset.

### NOTICE TO CONTRIBUTORS

Original articles are invited, provided they have not been published elsewhere. Notes of forthcoming events, meetings held, degrees conferred, staff appointments, and other local news are welcomed. The editorial committee reserves the right to make literary corrections.

Illustrations should always be supplied ready for reproduction. All re-touchings or other operations required will be charged to the author. Line drawings should be drawn in Indian ink. We regret that we must ask authors to pay for making blocks, if this is necessary.

J. W. ARROWSMITH LTD., WINTERSTOKE ROAD,
BRISTOL.

### Notes and News

Professor A. V. Neale has been appointed Heath Clark Lecturer (University of London) for 1961.

Mr. J. H. Peacock gave an Arris and Gale Lecture at the Royal College of Surgeons on 22nd March.

### Examination Results

UNIVERSITY OF BRISTOL

*M.D.*: T. J. Butler (with Distinction), C. C. Downie, J. E. Tovey.

*Ph.D.*: P. Radford.

*M.B., Ch.B.*

| | |
|---|---|
| Aubee, R. I. A. A. | Mayne, Madeline, D. |
| Benson, J. A. G. | Randell, D. T. H. |
| Duff, G. C. | Sibbald, Dorothy M. I. |
| Haskins, H. W. | Stayte, D. J. |
| Heighton, D. R. M. | Ugwunze, M. |
| Hill, J. P. A. | Undugodage, R. J. |
| Hodge, R. C. | Davies, K. R. |
| Lockett, G. W. E. | Kerr, G. |

### Appointments

P. A. James, M.B.(BRISTOL), F.R.C.S., thoracic surgeon (specialist), Uganda.

R. A. Sharpe, M.B.(BRISTOL), M.O. Bahamas.

*UNITED BRISTOL HOSPITALS*

*DECEMBER* 1959 *TO FEBRUARY* 1960

| *Name* | *Appointment* | *Formerly* |
|---|---|---|
| CATES, JOSEPH ELMHIRST, M.D., F.R.C.P. | Consultant Physician to the United Bristol Hospitals and the South Western Regional Hospital Board in the Bristol Clinical Area | Lecturer in Medicine, and Assistant Physician, United Bristol Hospitals. |
| GUPTA, P. B., M.B., B.S., F.R.C.S., M.CH.(ORTH.) | Senior Orthopaedic Registrar | Resident Orthopaedic Registrar, Norfolk and Norwich Group of Hospitals. |
| NESLING, A. E., M.B., CH.B. | Registrar in Anaesthetics | Registrar in Anaesthetics, City General Hospital Sheffield. |
| WALDRON, B. LE G., M.B., B.CH., D.A. | Registrar of Anaesthetics | Senior House Officer in Anaesthetics, United Bristol Hospitals. |
| WINSTONE, A., B.D.S., L.D.S.R.C.S. | Registrar in Dental Surgery | Dental Officer in the Royal Navy. |

Mr. J. S. Cooper, B.D.S., has been accorded honorary status as Registrar in the United Bristol Hospitals during the tenure of his appointment as Lecturer in Dental Surgery to the University of Bristol.

### *SOUTH WESTERN REGIONAL HOSPITAL BOARD DECEMBER, 1959 TO FEBRUARY, 1960*

| *Name* | *Appointment* | *Formerly* |
|---|---|---|
| | ***BRISTOL*** | |
| RODDIE, R. K., M.B., CH.B., B.A.O., F.R.C.S. | Consultant E.N.T. Surgeon, to the Bristol Clinical Area | Senior Registrar, E.N.T. Belfast Group of Hospitals. |
| FOSTER, G. S., M.D., B.S.(LOND.), M.R.C.O.G. | Consultant Obstetrician and Gynaecologist to the Weston-Super-Mare Group of Hospitals. | Senior Registrar in Obstretrics and Gynaecology, the United Cardiff Hospitals. |
| WILSON, F., M.B., B.S. (LOND.), F.F.A.R.C.S., | Temporary appointment of Consultant Anaesthetist, Southmead Hospital, Bristol. | Senior Registrar and Lecturer in Anaesthesia, University of Liverpool. |
| | ***BATH*** | |
| COLLEY, J., B.SC.(LOND.), M.B., B.S.(LOND.) | Medical Registrar to the Bath Group of Hospitals. | Clinical Officer in Medicine, BMH, Cyprus (Captain R.A.M.C.) |
| | ***SOUTH SOMERSET*** | |
| LEIGH, H. I., M.B., CH.B.(BRISTOL), F.R.C.S. (EDIN.) | Re-appointed Surgical Registrar, Musgrove Park Hospital, Taunton. | Surgical Registrar, Musgrove Park Hospital. |
| | ***NORTH GLOUCESTERSHIRE*** | |
| CHAMBERS, R., M.B., B.S. (DURHAM), D.P.M. | Assistant Psychiatrist Horton Road and Coney Hill Group of Hospitals. | Senior Registrar, Newcastle General Hospital. |
| LEWIS, J. W., M.B., B.S.(LOND.), D.(OBST.) R.C.O.G. | Re-appointed Clinical Assistant in Gynaecology, Stroud General Hospital (1 session). | In General Practice in Nailsworth. |
| GOMPERTZ, R. M. H., M.B., B.S.(LOND.), D. (OBST.) R.C.O.G., D.C.H. | Clinical Assistant in Paediatrics, Cheltenham (4 weekly sessions). | Is in general practice in Cheltenham. |
| DUNBAR, P. G., M.B., B.S.(LOND.), D.A., D. (OBST.) R.C.O.G. | General Practitioner Anaesthetist, Devizes Hospital (1 session). | In general practice in Devizes. |
| | ***DEVON AND EXETER*** | |
| HATCHER, G. W., M.A. (OXON.), M.B., B.S., D.R.C.O.G. | Clinical Assistant in General Medicine, Tiverton and District Hospital, (3 sessions). | Junior partner in general practice in Tiverton. |
| COOPER, R.A., M.B., B.S. (LOND.) | Temporary General Practitioner Anaesthetist, Bideford Hospital. (half-days per week). | In General Practice in Bideford. |
| SOLAN, M.J., M.B., B.S. (LOND.) | Surgical Registrar, South Devon and East Cornwall Hospital, Plymouth. | |
| O'ROURKE, D. A., M.B., B.S.(AUST.), F.R.C.S. (EDIN. & ENG.) | Surgical Registrar, South Devon and East Cornwall Hospital, Plymouth. | Senior Surgical Registrar, St. Institute of Urology, St. Paul's. |
| BHONSLE, R. S., M.B., B.S.(BOMBAY) | E.N.T. Registrar South Devon and East Cornwall Hospital, Plymouth. | Senior House Surgeon, Victoria Hospital, Worksop. |
| | ***CORNWALL*** | |
| SPENCER, R. W., M.B., CH.B., M.R.C.P. | Consultant Pathologist to the West Cornwall Clinical Area. | Senior Registrar, Lecturer in the Department of Chemical Pathology, University of Leeds. |

